# Modified Laminar Bone in *Ampelosaurus atacis* and Other Titanosaurs (Sauropoda): Implications for Life History and Physiology

**DOI:** 10.1371/journal.pone.0036907

**Published:** 2012-05-16

**Authors:** Nicole Klein, P. Martin Sander, Koen Stein, Jean Le Loeuff, Jose L. Carballido, Eric Buffetaut

**Affiliations:** 1 Steinmann Institute of Paleontology, University of Bonn, Bonn, Germany; 2 Musée des Dinosaures, Espéraza, France; 3 Museo Paleontologico Egidio Feruglio, Trelew, Argentina; 4 Centre National de la Recherche Scientifique, Paris, France; Raymond M. Alf Museum of Paleontology, United States of America

## Abstract

**Background:**

Long bone histology of the most derived Sauropoda, the Titanosauria suggests that titanosaurian long bone histology differs from the uniform bone histology of basal Sauropoda. Here we describe the long bone histology of the titanosaur *Ampelosaurus atacis* and compare it to that of basal neosauropods and other titanosaurs to clarify if a special titanosaur bone histology exists.

**Methodology/Principal Findings:**

*Ampelosaurus* retains the laminar vascular organization of basal Sauropoda, but throughout most of cortical growth, the scaffolding of the fibrolamellar bone, which usually is laid down as matrix of woven bone, is laid down as parallel-fibered or lamellar bone matrix instead. The remodeling process by secondary osteons is very extensive and overruns the periosteal bone deposition before skeletal maturity is reached. Thus, no EFS is identifiable. Compared to the atypical bone histology of *Ampelosaurus*, the large titanosaur *Alamosaurus* shows typical laminar fibrolamellar bone. The titanosaurs *Phuwiangosaurus*, *Lirainosaurus*, and *Magyarosaurus*, although differing in certain features, all show this same low amount or absence of woven bone from the scaffolding of the fibrolamellar bone, indicating a clear reduction in growth rate resulting in a higher bone tissue organization. To describe the peculiar primary cortical bone tissue of *Phuwiangosaurus, Ampelosaurus*, *Lirainosaurus*, and *Magyarosaurus*, we here introduce a new term, “modified laminar bone” (MLB).

**Conclusions/Significance:**

Importantly, MLB is as yet not known from extant animals. At least in *Lirainosaurus* and *Magyarosaurus* the reduction of growth rate indicated by MLB is coupled with a drastic body size reduction and maybe also a reduction in metabolic rate, interpreted as a result of dwarfing on the European islands during the Late Cretaceous. *Phuwiangosaurus* and *Ampelosaurus* both show a similar reduction in growth rate but not in body size, possibly indicating also a reduced metabolic rate. The large titanosaur *Alamosaurus*, on the other hand, retained the plesiomorphic bone histology of basal neosauropods.

## Introduction

### Long Bone Histology of Sauropoda

The histology of fossil tetrapod bones has proven to be a rich source of paleobiological and evolutionary information [1]–[4], particularly for groups that have no living relatives or in which the living relatives occupy a fundamentally different ecological niche. This is particularly true for sauropod dinosaurs, the largest land-living animals ever [4], [5], for which a large amount of histological data have become available in recent years [4], [6], [7]. Sauropod long bone histology so far has been viewed as rather uniform [4], [6], allowing broad comparisons between taxa [6]. However, bone histology has been less forthcoming in providing characters for phylogenetic analysis, because phylogeny is not the controlling factor in bone histogenesis [8], [9].

**Figure 1:**
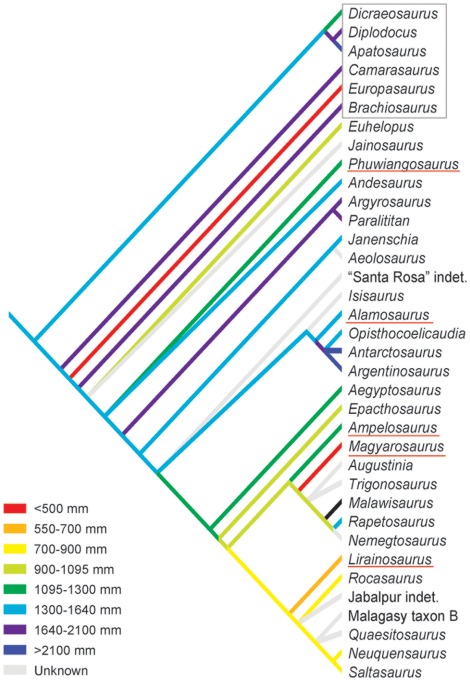
Phylogenetic relationships of the sampled titanosaur taxa (underlined in red) and outgroup taxa (black box) and body size evolution in Titanosauria. For the reconstruction of body size evolution, we used femur length as a proxy for body size and optimized it on the only phylogeny that includes all the taxa discussed in this study [10]. *Europasaurus* was inserted into this phylogeny as a basal macronarian [20]. *Magyarosaurus*, *Lirainosaurus*, and *Europasaurus* show autapomorphic size decrease, i.e., dwarfing. *Phuwiangosaurus* also has a slightly reduced body size compared to the stem line, while *Ampelosaurus* shows a slight phylogenetic size increase. Character optimization analysis was performed in TNT [41].

The most morphologically derived sauropods, the Titanosauria, remain poorly sampled (Fig. 1) because of the relative incompleteness of their remains and the difficulties in their taxonomical assignment. This contrasts with their great diversity and dominance in Cretaceous faunas, particularly on the southern continents [10]. Recently, evidence has accumulated [11]–[13] that titanosaurian long bone histology may be different from that of the more basal neosauropods, i.e., diplodocoid and basal macronarian sauropods of the Jurassic. While some of these differences may be attributed to evolutionary size changes related to island habitats (*Magyarosaurus* [13]; *Lirainosaurus* [12]), the case is less clear-cut in the limited number of other taxa (*Phuwiangosaurus* [11]) that have been studied. Contrary to those smaller sized forms, the large titanosaur *Alamosaurus* shows a more typical bone tissue type comparable to that of the Jurassic sauropods [7]. It is in this context that our contribution focuses on a growth series of the derived titanosaur *Ampelosaurus atacis* Le Loeuff, 1995 [14] from the Late Campanian-Early Maastrichtian of southern France [15].

The differences between basal neosauropods and some titanosaurs are related to the scaffolding of the fibrolamellar bone deposited in the long bones of those sauropods. The typical fibrolamellar bone of basal Neosauropoda is a 3D structure which shows a sequential deposition of different bone types to form a complex tissue. In a first step, a scaffolding of woven (or fibrous) bone matrix is deposited fast around a large vascular canal, and only later is the vascular canal filled in centripetally by lamellar bone. This infill is then called a primary osteon. Fibrolamellar bone is highly vascularized, and its vascular system is dominated by circumferential vascular canals wherefore the tissue is also called laminar fibrolamellar bone, or laminar bone for short.

By describing the long bone (humerus and femur) histology of *Ampelosaurus atacis* in detail and reviewing the known histology of other titanosaurs (Fig. 1), we would like to address the following questions: Is there such a thing as special titanosaurian bone histology, as proposed by Company [12]? Can all the peculiarities of titanosaurian bone histology be explained by evolutionary body size reduction? What is the role of island dwarfing in shaping titanosaur bone histology? What are the implications of titanosaurian bone histology for life history, physiology, and evolution of the group? As always, a comparative histological approach is necessary, comparing not only different taxa of titanosaurian and non-titanosaurian sauropods (Fig. 1) but also the same histologic ontogenetic stages (HOS of Klein and Sander [6]). At this point we would like to emphasize again the importance of sampling homologous points in the long bones of the different sauropod taxa [4], [6], [16] without which comparisons become difficult, if not impossible.

### Previous Work on Sauropod Bone Histology

#### Bone histology of Diplodocoidea and basal Macronaria

Because primary bone tissues and patterns of remodeling by secondary osteons are very similar in all studied Diplodocoidea and basal Macronaria [1], [2], [4], [6], [16], [17], [18], Klein and Sander [6] erected histological ontogenetic stages (HOS) for these taxa and correlated them with biological ontogenetic stages (BOS) [4]. The HOS were defined on the basis of changes in the principally laminar fibrolamellar bone tissue. These changes mainly concern the type of primary bone tissue (i.e., the amount of woven bone matrix, parallel-fibered bone matrix, and lamellar bone matrix in the fibrolamellar complex), organization of the vascular system, the degree of vascularization, the presence and degree of development of primary osteons, the presence of growth marks, and the appearance of an external fundamental system (EFS). A further important characteristic is the occurrence and density of secondary osteons [6]. Thus, each HOS for Diplodocoidea and basal Macronaria is defined by a certain bone tissue type, the absence or presence of growth marks and an EFS, and by the degree of remodeling by secondary osteons [6].

Bone histology of humeri and femora of basal Macronaria is similar to that of most other basal Neosauropoda (mainly Diplodocoidea). They share a thick cortex which consists of large amounts of laminar fibrolamellar bone tissue. Later in ontogeny, the fibrolamellar bone tissue changes to lamellar zonal bone tissue, called an external fundamental system, convergent on the conditions in mammals (EFS; sensu Cormack [19]). The EFS is thought to indicate a growth plateau which indicates that maximum body size and skeletal maturity is reached in an individual. No kind of growth marks appear in those taxa before type E bone tissue is deposited (HOS 9 or 10; [6]), and growth marks usually remain rare until late ontogenetic stages, when a distinct slow down in growth rate occurs. Vascularization, and therefore growth rate, decreases gradually from young to fully grown individuals. Dense remodeling of the primary cortex by secondary osteons is characteristic of Diplodocoidea and basal Macronaria. Remodeling by secondary osteons starts in young adults (type D bone tissue, HOS 8) and continuously progresses to very old individuals, in which the entire cortex is made of dense secondary osteons ( = Haversian bone).

#### Bone histology of *Europasaurus* as described by Sander et al. [20]

*Europasaurus holgeri* is a dwarfed, basal macronarian from Kimmeridgian (Upper Jurassic) marine sediments of the Lower Saxony Basin in northern Germany [20]. Its long bone histology resembles that of large basal neosauropods and consists of laminar fibrolamellar bone with an EFS deposited in the outermost cortex of fully grown individuals. Remodeling by secondary osteons increases during ontogeny and is comparable to the pattern seen in basal Neosauropoda. *Europasaurus* differs from other basal neosauropods in having growth marks in the form of lines of arrested growth (LAGs) throughout its entire cortex, even in ontogenetically young individuals [20]. However, except for the occurrence of these LAGs and the therefore cyclical interruption of growth, the HOS defined for neosauropods are well applicable for *Europasaurus.* The authors hypothesized that the regular occurrence of growth marks in *Europasaurus* indicates that bone apposition rate was lower when compared to other basal Neosauropoda. Additionally, growth mark count suggests that the period of active growth was shortened. This implies that *Europasaurus*, which has a body length of only 6 m, reached its diminutive body size by a reduction in growth rate and a shortening of ontogeny [20]. The reduction in growth rate is indicated by the regular occurrence of lines of arrested growth, which are lacking in all large-bodied sauropods [4], [6], whereas the shortening of ontogeny is documented by comparing growth mark counts.

#### Bone histology of *Alamosaurus* as described by Woodward and Lehman [7]

Bone histology of *Alamosaurus sanjuanensis* from the Upper Cretaceous Javelina and Black Peaks formations in Big Bend National Park, Texas, was extensively studied from different bones of the skeleton by Woodward and Lehman [7]. Woodward and Lehman [7] described laminar fibrolamellar bone tissue for a humerus of 59% maximum length; in a humerus of 78% maximum length, most of the compact bone is composed of secondary osteons, but remains of primary bone show parallel-fibered bone tissue, and a femur of 81% maximum length is completely remodeled by secondary osteons. Woodward and Lehman [7] determined the bone tissue type for each bone and HOS following the definition of Klein and Sander [6]. Woodward and Lehman [7] did not find an EFS in any of their *Alamosaurus* samples, but they mentioned the possibility of not having sampled fully grown individuals. They found extensive remodeling by secondary osteons in *Alamosaurus* starting with HOS 7 [7], which is earlier in ontogeny than in Diplodocoidea where at HOS 7 secondary remodeling starts only scattered [6]. Finally, Woodward and Lehman stated that *Alamosaurus* bone histology largely resembles that of *Apatosaurus* as described by Curry [21].

#### Bone histology of *Phuwiangosaurus* as described by Klein et al. [11]

Humeri and femora of the medium-sized basal titanosaur *Phuwiangosaurus sirindhornae* from the Lower Cretaceous of Thailand were sampled by core drilling at the standardized sampling location [11]. In general, the bone tissue is continuously growing laminar fibrolamellar bone, typical for other sauropods. The samples of *Phuwiangosaurus* can be largely assigned to the HOS of Klein and Sander [6], [11], although bone histology of *Phuwiangosaurus* differs in some respects from the original definition of the HOS. For example, type D bone tissue and type E bone tissue have a much higher amount of parallel-fibered and lamellar bone matrix in the fibrolamellar complex when compared to more basal neosauropods. Furthermore, type G bone tissue is not developed, which means that none of the bones is completely or nearly completely remodeled by secondary osteons. Large femora consisting of type E or F bone tissue do not show strong remodeling by secondary osteons which is contrary to the condition in humeri. Growth marks may occur in the outer cortex of both, humeri and femora, but none of the samples shows an EFS. No complete shaft cross sections are known from *Phuwiangosaurus* bones, but based on the observation of deep drill cores reaching the inner cortex of the opposite side of the shaft, the medullary region is filled by secondary trabeculae.

Klein et al. [11] concluded that *Phuwiangosaurus* had a lower growth rate than diplodocoids and basal macronarians. Despite covering a great size range, the *Phuwiangosaurus* sample may not include very large and old ( = fully grown) individuals, because a fully remodeled cortex and an EFS are missing. Differences in the degree of remodeling between humeri and femora may be explained by biomechanical factors such as strains and forces during limb growth and locomotion, or they could indicate lower apposition rates of the cortical bone in the humerus compared with the femur because of the smaller midshaft diameter of the humerus.

#### Bone histology of *Lirainosaurus* as described by Company [12]

*Lirainosaurus astibiae* from the Upper Cretaceous of northern Spain is a small (8–10 m) titanosaur related to the Eutitanosauria [22] and possibly also to the South American Saltasaurinae [10]. The histological study of eleven long bones revealed that *Lirainosaurus* grew with laminar fibrolamellar bone tissue, which was irregularly interrupted by growth marks [12]. Intense secondary remodeling started early in ontogeny and tended to replace the entire primary bone tissue [12]. *Lirainosaurus* has a thin bone wall in the femur, with a laterally thicker cortex when compared to the anterior and posterior bone sides [12]. The narrow medullary cavity is almost filled with secondary cancellous tissue and is surrounded by a medullary region containing trabeculae [12]. Mainly on the basis of remodeling by secondary osteons, “young adult individuals”, which equal HOS 11 of Klein and Sander [6], and “adult individuals” were distinguished. According to Company [12], no EFS was developed in the outer cortex of *Lirainosaurus* long bones. Company [12] concluded that *Lirainosaurus* attained its smaller size compared to typical sauropods by reducing the rate of primary periosteal apposition and by developing an extensive secondary remodeling well before adult size was reached. He explained the bone histological characteristics of *Lirainosaurus* as peramorphosis by pre-displacement and sees a reversal of the accelerated pattern of bone deposition normally typical for the sauropod lineage [12]. Company [12] follows other authors and finds the small body size of *Lirainosaurus* best explained by insular dwarfing.

#### Bone histology of *Magyarosaurus* as described by Stein et al. [13]

*Magyarosaurus dacus* is a dwarfed titanosaur from Maastrichtian terrestrial sediments of Romania. The long bone histology of *Magyarosaurus* is unique among sauropods, because even the smallest individuals of 45% maximum size show nearly complete replacement of the primary cortex by secondary osteons. The well-defined medullary cavity is small and surrounded by cancellous bone. The cortex is thick. The primary organization of the bone tissue is laminar fibrolamellar bone, which mainly consists of parallel-fibered and lamellar bone matrix and has only a minimal amount of woven bone matrix. *Magyarosaurus* did not have an EFS developed in any sampled individual. Due to the extensive remodeling and the high amount of slow-growing bone matrix in the fibrolamellar complex, the HOS was not strictly applicable. Also, Stein et al. [13] erected an additional histological ontogenetic stage (HOS 14) to obtain a higher ontogenetic resolution. In two samples of *Magyarosaurus*, lines of arrested growth are visible (humerus V13492, humerus R 1195), although they are vague and cannot be traced along the complete circumference of the section. Stein et al. [13] concluded that the intense remodeling of the *Magyarosaurus* cortex suggests an extremely reduced growth rate compared to basal neosauropods. Stein et al. [13] interpreted the bone histology and diminutive body size of *Magyarosaurus* as a result of insular dwarfism.

## Materials and Methods

### Ampelosaurus

Growth series of humeri and femora assigned to the Late Cretaceous (Late Campanian – Early Maastrichtian) titanosaur *Ampelosaurus atacis* were sampled. New finds of *Ampelosaurus* revealed that the maximum known femur length (FL) (ca. 1100 mm, MDE field no. C3-02-172) as well as the largest femur sampled in our study are much larger than the FL cited for *Ampelosaurus* in the literature (802 mm [23]). This also necessitated a revision (Fig. 1) of the phylogenetic optimization of body size of titanosaurs by Stein et al. [13]. The current size optimization shows that *Ampelosaurus* slightly increased in size compared to the ancestral condition. However, the largest humerus sampled is from a distinctly smaller individual than the largest femur known.

**Figure 2:**
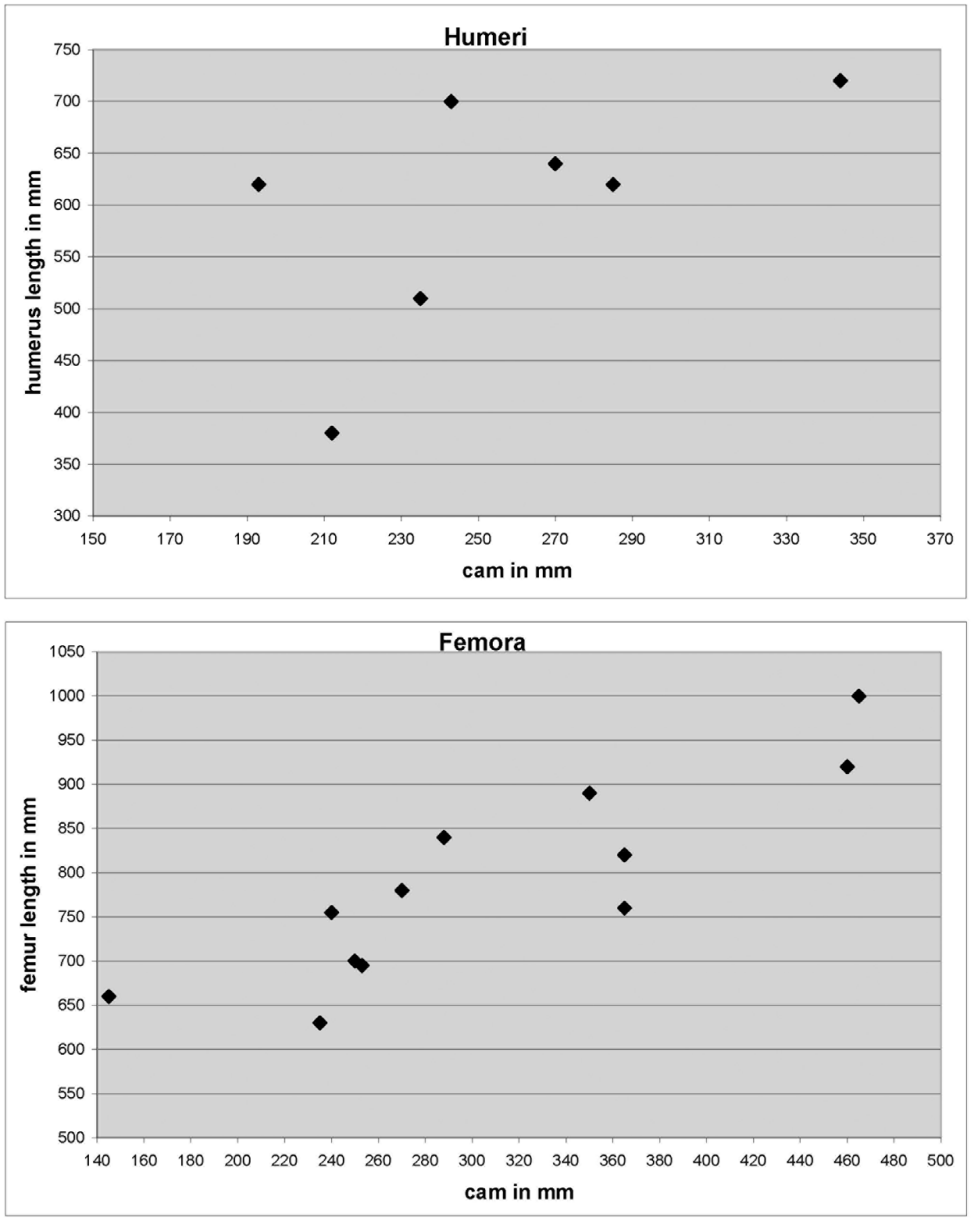
Circumference at midshaft (cam) plotted versus bone length (in millimeter) of humeri and femora of *Ampelosaurus atacis* from the locality of Bellevue from the Upper Aude Valley. A) Femora. B) Humeri. The plot suggests that there are two morphotypes, because humeri and femora of a similar length form two groups in terms of circumference. A gracile and a more robust type is thus suggested by differences around 100 mm in circumference at midshaft. This graph also includes bones which were not sampled.

While femur length is generally a good proxy for body size in sauropods [4], [23], the measurement is difficult to obtain in the sometimes poorly preserved *Ampelosaurus* material. This also applies to the humeri sampled here. In addition, the correlation between femoral or humeral length and circumference is not very strong (Fig. 2, regression line: R^2^ = 0.7579 for femora, R^2^ = 0.3365 for humeri) compared to other sauropods [24]. This is mainly because humeri and femora of *Ampelosaurus*, each of a similar bone length, can differ in circumference at midshaft by around 100 mm (Table 1, Fig. 2), possibly documenting a gracile and a more robust morphotype [25]. Therefore, we use minimal midshaft circumference as a proxy for body size here, well aware that this is a relatively poor predictor of length in both femora and humeri but a good proxy for body mass. Sampled humeri ranged in midshaft circumference from 130 mm to 320 mm, whereas the size range of femora was smaller, with 235 mm to 480 mm (Table 1). Although we chose to use shaft circumference as a proxy for body size, we recognize that femur and humerus lengths calculated from circumference may have to be used in future comparative work instead of circumferences.

**Table 1:** Sampled material of *Ampelosaurus atacis* from Bellevue locality (MDE C3) and of the titanosaurs from north of Narbonne (Cru).

Humeri	Length	Cam	Sample	Bone side	Comment
C3-977	>190	130	cross section	anteriorposterior	diaphyseal frgm.
C3-270	>180	170	cross section	anteriorposterior	diaphyseal frgm.
C3-1506	620	195	thin section	anterior	complete bone
C3-175	700	243	bone fragment	posterior	complete bone
C3-602	>350	250	bone fragment	posterior	incomplete bone
Cru-1723	>400	250	bone fragment	anterior	diaphyseal frgm.
C3-238	>340	270	bone fragment	anteriorposterior	diaphyseal frgm.
C3-1189	>320	310	thin section	anterior	diaphyseal frgm.
Cruzy-1	>600	nm	bone fragment	posterior	incomplete bone
**Femora**					
Cru-2	630	235	thin section	posterior	complete bone
Cru-3	>480	270	cross section	anteriorposterior	diaphyseal frgm.
C3-1182	695	253	thin section	anterior	complete bone
C3-708	>400	260	thin section	posterior	incomplete bone
C3-203	780	270	thin section	anterior	complete bone
Cru-6	>620	275	cross section	anteriorposterior	diaphyseal frgm.
C3-527	680	280	bone fragment	anterior	complete bone
C3-261	840	288	thin section	anterior	complete bone
C3-638	>605	285	bone fragment	posterior	incomplete bone
Cru-4	>550	285	bone fragment	anteriorposterior	incomplete bone
C3-143	>420	348	bone fragment	median	incomplete bone
Cru-5	890	350	thin section	posterior	complete bone
C3-1239	>556	350	thin section	anterior	incomplete bone
C3-582	>635	37.5	bone fragment	posterior	incomplete bone
C3-78	>1000	465	thin section	posterior	incomplete bone
C3-174	>690	480	thin section	anterior	diaphyseal frgm.

Bones are arranged by midshaft circumference because bone length or reliable reconstructions thereof were not available for many specimens (see Material and Method section). Measurements all given in millimeters. Cam = Circumference at midshaft.

Sampled bones are stored in the Musée des Dinosaures, Espéraza, France (labelled as MDE; note: C3 is the code for the locality of Bellevue) or in the local museum of Cruzy, France (labelled as Cru). Bones from the MDE originate from the Bellevue locality in the Upper Aude Valley. The excavation site is located near the small village of Campagne-sur-Aude, which is 3 km southwest of Espéraza. The Bellevue locality has mainly produced disarticulated bones [15] although a partially articulated titanosaur skeleton is under study. Recent studies have questioned the occurrence of only one titanosaur taxon from this locality [25]. However, the bone bed character of the locality, the preservation of bones, and the uniform morphology of sauropod/titanosaur long bones limits exact taxonomical assignment of isolated humeri and femora.

The bones labelled “Cru” originate from three localities: Cruzy, Montouliers, and Massecaps, all located north of Narbonne and not in the Aude Valley. Stratigraphical age and sediments are similar to the titanosaur locality in the Aude Valley, possibly representing the same historical environment [26]. Therefore, those bones have been preliminarily assigned to *Ampelosaurus atacis* (Buffetaut pers. obs.). However, ongoing morphological studies indictae that the material from Massecaps (Cru-1723, Cru-4) did not belong to *A. atacis* but to a new titanosaur (Buffetaut and Le Loeuff pers. obs.). This might be also the case for the other material labelled as “Cru”. However, long bone histology of those samples did not differ from that of *A. atacis* and can therefore be included in the current study.

The sample location was always in the midshaft region, to gather the most complete record of the appositional growth phase and for comparability with samples from other studies. The standard sampling location for humeri is the posterior bone side of the midshaft, and for femora it is the anterior bone side of the midshaft [4], [16], [27]. However, due to preservation of *Ampelosaurus* bones it was sometimes necessary to sample the opposite side of the shaft. As described in the “Results” section, bone tissue varies between the anterior and posterior bone side of humeri and femora in *Ampelosaurus*. Humeri show posteriorly an older bone tissue and therefore a later histological ontogenetic stage and a higher degree of remodeling by secondary osteons than anteriorly (see Table S1). In femora, the reverse is true. While to be expected, the difference between the anterior and the posterior side of the bone has not been described in any detail in other sauropod long bones previously. For comparing the *Ampelosaurus* samples with those from other sauropods, we use only the histology at the standardized location, even when the entire cross section is available.

### Alamosaurus

**Figure 3:**
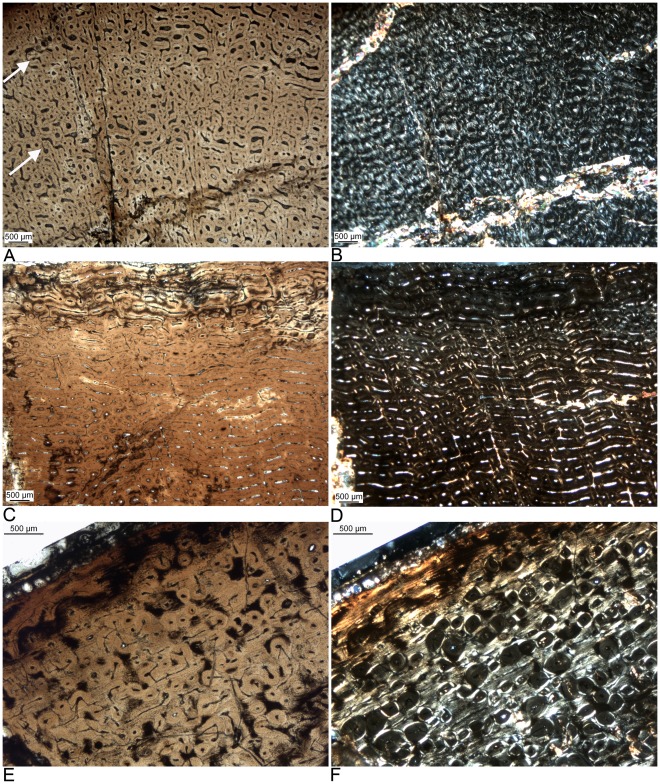
Histological details of the humeral cortex of a growth series of the derived titanosaur *Alamosaurus sanjuanensis* from the Upper Cretaceous Javelina Formation, Texas, USA. The bones tissues in this growth series of the large titanosaur *Alamosaurus* are identical to those of large diplodocoid and basal macronarian sauropods. The plane of sectioning is perpendicular to bone long axis, and the direction of bone apposition is towards the top of the images. **A**) Typical laminar fibrolamellar bone tissue of a young individual (HOS 4; TMM 45600-1; 460 mm long, 31% max. size) in normal light. Note the absence of LAGs but the subtle modulations in vascularization (arrows). **B**) Same view in polarized light. Note the dominance of the scaffolding of woven bone in the fibrolamellar complex. **C**) Typical laminar fibrolamellar bone tissue of a young adult individual (HOS 7; TMM 43600-2; 915 mm long, 61% max. size). Note the strictly circumferential arrangement of the vascular canals. **D**) Same view in polarized light. Note the primary osteons in the scaffolding of woven bone. **E**) Incipient EFS in the outermost cortex and scattered secondary osteons in laminar fibrolamellar bone of a fully grown individual (HOS 12; TMM uncat.; 1350 mm long, 90% max. size). **F**) Same view in polarized light. Note the highly birefringent parallel-fibered bone of the EFS and the woven to parallel-fibered scaffolding of the fibrolamellar bone in the deeper cortex, between the secondary osteons. The high amount of parallel-fibered bone in the scaffolding of the fibrolamellar bone is typical in late ontogenetic stages in diplodocoid and basal macronarian dinosaurs [6] as well as for *Alamosaurus* but occurs already in early ontogenetic stages in *Ampelosaurus*.

Four humeri of *Alamosaurus sanjuanensis* from the Upper Cretaceous Javelina Formation in Big Bend National Park, Texas, housed in the TMM were sampled by core drilling (Table 2; Fig. 3) according to the methods described by Sander [16] and Stein and Sander [27]. The humeri represent a small growth series and were sampled to supplement the data set of Woodward and Lehman [7].

**Table 2:** Bone histological sample of *Alamosaurus sanjuanensis* stored in the SIPG.

bone	coll. no.	bone length	circumference	HOS	Percentage max. size
humerus	TMM 45600-1	460 mm	175 mm	HOS 4	31%
humerus	TMM 43600-2	915 mm	375 mm	HOS 7	61%
humerus	TMM 43090-1	1300 mm	595 mm	HOS 10	87%
humerus	TMM uncataloged	1350 mm	650 mm	HOS 12	90%

The percentage value for maximum size for each sample was calculated on the basis of TMMM 41541-1 (humerus, 1503 mm) as described by Lehman and Coulson (2002).

Sampling was by core drilling, extraction of bone fragments, and cross sectioning at midshaft. The coring method used here has been described in detail before [4], [27], [28]. The cores and bone pieces were embedded in epoxy resin and cut perpendicular to the long axis of the bone, which is also perpendicular to the growth direction. Half of the sample was processed into a standard petrographic thin section and the other half into a polished section. Thin sections were examined by standard light microscopic techniques (normal transmitted light, polarized light) with a Leica DMLP compound microscope (1.6x to 40x objective lenses). Terminology follows Francillon-Vieillot et al. [29] and Klein and Sander [6].

### Institutional Abbreviations

local museum of Cruzy, France

Musée des Dinosaures, Espéraza, France

Texas Memorial Museum, Austin, Texas, North America

## Results

### Long Bone Histology of *Ampelosaurus Atacis*

#### Primary cortex

**Figure 4:**
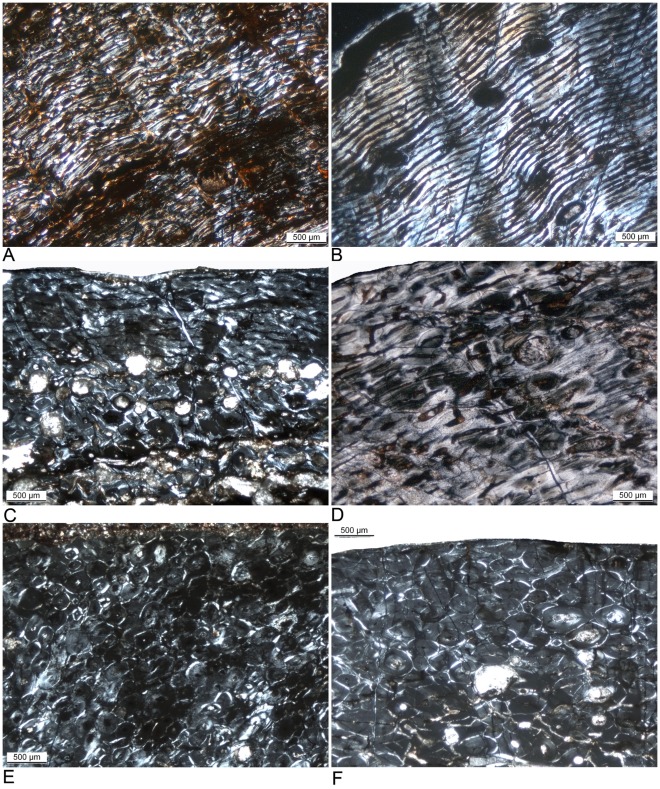
Histological details of humerus bone tissue of *Ampelosaurus atacis* from Bellevue locality (MDE C3) and from a titanosaur north of Narbonne (Cru), all from the Maastrichian of South France. **A**) Modified laminar bone at the anterior bone side of humerus C3-270 (cam 170 mm). The vascular canals are wide open, and primary osteons had not yet developed. The scaffolding of the fibrolamellar bone consists largely of parallel-fibered and lamellar bone matrix. This bone tissue represents HOS 7. **B**) Modified laminar bone at the posterior bone side of humerus C3-270 (cam 170 mm). The vascular canals are still distinctly open. Laminar organization is greater when compared to the anterior bone side. Primary osteons had partially developed. The scaffolding of the fibrolamellar bone consists of parallel-fibered and lamellar bone matrix. This bone tissue represents HOS 9. **C**) Modified laminar bone at the anterior bone side of humerus C3-1506 (cam 195 mm, humerus length is 620 mm). Primary bone tissue is visible in the upper third of the cortex, whereas the inner part is remodeled by secondary osteons. The scaffolding of the fibrolamellar bone consists of parallel-fibered and lamellar bone matrix. Primary osteons are nearly filled in. This bone tissue represents HOS 11. **D**) Modified laminar bone of humerus Cru-1723 (cam 250 mm). The scaffolding of the fibrolamellar bone consists largely of lamellar bone matrix. The vascular density in this sample is still moderate with still open primary osteons. Most of the cortex is completely remodeled by secondary osteons. The outer cortex shows dense secondary osteons. This bone tissue represents HOS 12. **E**) The posterior bone side of humerus C3-602 (cam 250 mm) is completely remodeled by secondary osteons representing HOS 13. **F**) The anterior bone side of humerus C3-1189 (cam 310 mm) is completely remodeled by secondary osteons representing HOS 13.

**Figure 5:**
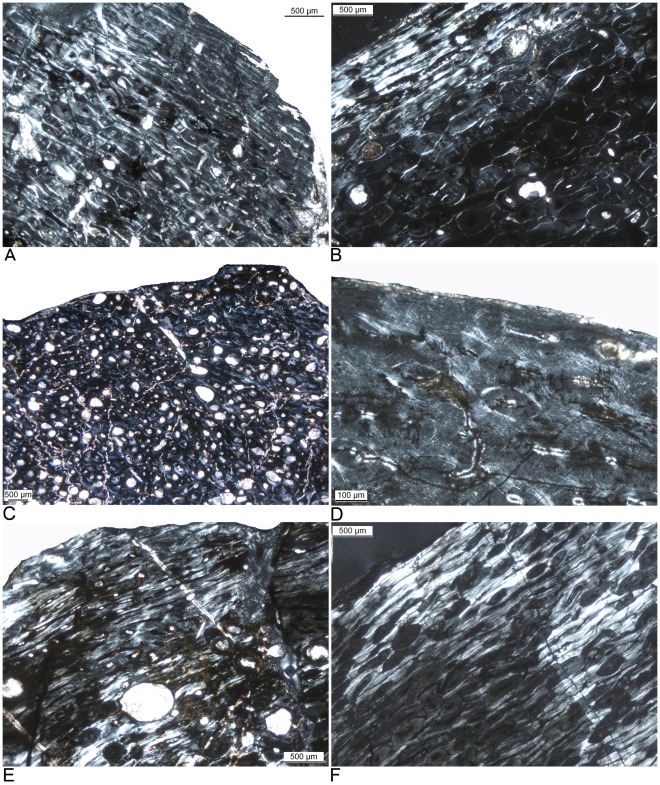
Histological details of femur bone tissue of *Ampelosaurus atacis* from Bellevue locality (MDE C3) from the Maastrichian of South France. **A**) Modified laminar bone at the anterior side of femur C3-1182 (cam is 255 mm, femur length is 695 mm). The scaffolding of the fibrolamellar bone consists largely of parallel-fibered bone matrix. The vascular canals are still open. Primary osteons had partially developed. Dense remodeling occurs up to the middle cortex, and there are scattered secondary osteons in the outer cortex. This bone tissue represents HOS 11. **B**) Modified laminar bone on the anterior side of femur C3-203 (cam 270 mm, femur length is 780 mm). The scaffolding of the fibrolamellar bone consists largely of lamellar bone matrix. The vascular canals are still open, and primary osteons had partially developed. Dense remodeling occurs up to the outer cortex. This bone tissue represents HOS 12. **C**) Modified laminar bone on the anterior side of femur C3-527 (cam 280 mm, femur length is 680 mm). The primary cortex is completely remodeled by secondary osteons. This bone tissue represents HOS 13. **D**) Modified laminar bone on the anterior bone side of femur C3-261 (cam 288 mm, femur length is 840 mm). The vascular canals are nearly filled in. Primary osteons had only poorly developed. The scaffolding of the fibrolamellar bone consists largely of parallel-fibered bone matrix. This bone tissue represents HOS 12. **E**) Modified laminar bone on the anterior side of femur C3-1239 (cam 350 mm). The vascular canals are still open. Primary osteons are well developed. The scaffolding of the fibrolamellar bone consists of parallel-fibered and lamellar bone matrix. This bone tissue represents HOS 11. **F**) Modified laminar bone on the anterior side of femur C3-78 (cam 465 mm). The vascular canals are still open. Primary osteons are well developed. The scaffolding of the fibrolamellar bone consists laregly of lamellar bone matrix. Secondary osteons are only scattered throughout the cortex. This bone tissue represents HOS 11.

The primary bone tissue of all sampled *Ampelosaurus* bones shows principally the typical laminar organization and a 3D structure (scaffolding) of bone deposition similar to the fibrolamellar bone of Diplodocoidea and basal Macronaria (as described before and by, e.g., [4], [6], [7], [11], [12], [16], [17], [30]). However, in one aspect, the primary cortical bone tissue of *Ampelosaurus atacis* differs largely from that of more basal neosauropod dinosaurs (Fig. 4, 5), because already at an early ontogenetic stage the scaffolding of the fibrolamellar bone is partially laid down as parallel-fibred and lamellar bone matrix instead of woven bone matrix (Table S1, Fig. 4, 5). In adult but still growing individuals, no woven bone matrix was deposited at all in the primary cortex (Fig. 4, 5).

**Figure 6:**
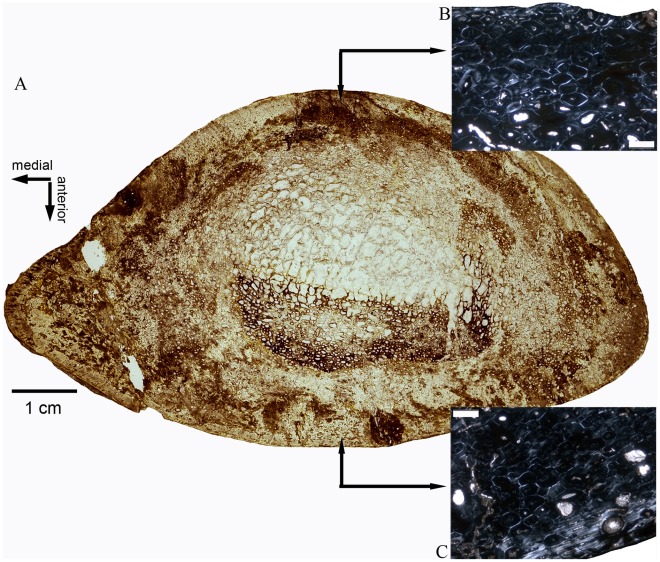
Overview (A) and enlargements (B, C) of a cross section of an *Ampelosaurus atacis* humerus (MDE C3-328, cam 270 mm) from Bellevue locality. On the posterior side (B), the humerus shows an “older” bone tissue (HOS 13) than at the anterior side (C) (HOS 12). Scale bars in B and C equals 0.5 mm.

Osteocytes are in most places numerous, although often flattened. In many samples the outermost cortex is partially or completely absent due to poor preservation. On the basis of the cross sections, the posterior bone side of the humeri always shows “older” bone tissue (i.e., a higher HOS) than the anterior side (Fig. 6). Thus, the primary cortex is more organized, less vascularized, and more remodeled when compared with the bone tissue at the anterior bone side (Table S1; Fig. 6). The opposite situation seems to be true for femora, although our study includes only one informative femur cross section.

#### Vascular organization and vascular density

The vascular density is generally higher in early histological ontogenetic stages (Fig. 4, 5) and decreases progressively with increasing ontogenetic age, as was described before for other sauropods [6]. However, vascular density remains relatively high with still open vascular canals in the outer cortex even when the fibrolamellar bone has a primarily lamellar bone scaffolding. On the basis of the cross sections, the posterior bone side of the humeri always show a lower vascular density when compared with the anterior bone side (Table S1; Fig. 4, 5), as noted above. In femora, the anterior bone side shows a lower vascular density. The organization of vascular canals is laminar in most samples, but some show a less laminar organization and a higher amount of longitudinal vascular canals (Table S1). In one humerus cross section (MDE C3-270), the vascular canals are diagenetically widened. In some samples of a similar HOS, primary osteons are well developed, but only poorly developed in others (Table S1). This means that no distinct centripetal infilling of the vascular canal by lamellar bone matrix is visible. This infilling is then hard to distinguish from the initially laid down framework of lamellar matrix.

#### Growth marks and EFS

In none of our samples are growth marks of any kind or an EFS developed (Table S1) despite sufficient preservation of the outer bone surface. However, since we did not sample the largest individuals assigned to *Ampelosaurus* (Le Loeuff pers. obs.), we cannot exclude the possibility that these preserve an EFS. In addition, an EFS may have been present in some of the bones in our sample but may have been obliterated by remodeling (see below).

#### Remodeling by secondary osteons

Remodeling by secondary osteons is common in the current sample, with only three exceptions. Secondary osteons are not developed in MDE C3-977 and MDE C3-270, which, on the basis of their primary bone tissue, represent early HOS (Fig. 4A, B; Table 3, Table S1). The femur sample Cru-3, which is assigned to HOS 11 (Table 3), shows only a few scattered secondary osteons throughout the entire cortex and cross section. All other *Ampelosaurus* samples show dense secondary osteons in the inner and middle cortex with scattered ones in the outer cortex, or they are already completely or nearly completely remodeled by secondary osteons (Table S1). In the humeri, the process of remodeling by secondary osteons is faster on the posterior bone side than on the anterior bone side (Fig. 6); in femora it is the other way around (Table S1). In one sample (Cru-4), the secondary osteons have an untypical, elongated diamond-shaped form, although the sampling plane is the same in all samples. This suggests that these secondary osteons are not oriented strictly longitudinally but at some angle to the bone long axis, which is atypical for Neosauropoda.

**Table 3:** Definition of the modified HOS for *Ampelosaurus atacis* from Bellevue locality (MDE C3) and the titanosaurs from north of Narbonne (Cru).

Bone tissue type	HOS	Sample (MDE)
type A and type B bone tissue are not known for *Ampelosaurus*	HOS 1–5	no sample available
cortex consists primarily of type C bone tissue with type D bone tissue laid down in the outer cortex	HOS 6	C3-977 (anterior)
cortex consists primarily of type D bone tissue while in the inner cortex remains of type C bone tissue can be preserved	HOS 7	C3-270 (anterior)
cortex consists primarily of type D bone tissue with type E bone tissue laid down in the outer cortex	HOS 8	C3-270 (posterior)
cortex consists primarily of type E bone tissue while in the inner cortex remains of type D bone tissue can be preserved	HOS 9	C3-977 (posterior),
cortex consists primarily of type E bone tissue with type F bone tissue laid down in the outer cortex	HOS 10	Cru-3 (posterior), C3-638 (posterior)
cortex consists primarily of type F bone tissue while in the inner cortex remains of type E bone tissue can be preserved dense remodeling	HOS 11	C3-1506; C3-1182, Cru-3 (anterior), C3-638 (anterior), C3-1239, C3-78
bone tissue as above but remodeling is more intensive	HOS 12	C3-1723, C3-238 (anterior), C3-203, C3-261, Cru-4 (posterior), C3-174
completely remodeled by secondary osteons	HOS 13	C3-175, C3-602, C3-238 (posterior), C3-1189, Cru-1; Cru-2, C3-708, C3-527, C3-143, Cru-4 (anterior), Cru-5, C3-582, Cru-6

The sequence of bone tissues for each HOS in *Ampelosaurus* and other Late Cretaceous titanosaurs from southern France is newly defined here to permit a better comparison with the HOS for Diplodocoidea and basal Macronaria of Klein and Sander (2008). See Table S1 for detailed histological information on each specimen.

#### Medullary region

A free medullary cavity does not exist in *Ampelosaurus*, but the medullary region consists completely of secondary cancellous bone (Fig. 6). The medullary region is relatively large in all samples (Table S1). In the triangular to oval humeral cross section of MDE C3-270 and MDE C3-238, the medullary region is more or less centered, and the primary cortex is consistently thin all around the cross section, except for the medial bone side where the primary cortex is thicker. In the oval cross section of humerus MDE C3-977, the medullary region is smaller and displaced towards the posteromedial bone side. The primary cortex is very thick along the anterolateral bone sides (more than three times when compared to the other bone sides). In both samples, the secondary trabeculae of the medullary region are partially replaced in the center by a postmortem modification in form of a hole that was filled with fine sediment during fossilization. These holes apparently were caused by some bone-mining organism such as arthropods. The round to oval cross sections of femora Cru-3 and Cru-6 show a relatively smaller medullary region when compared to the humerus cross sections (Table S1). Both medullary regions originally contained secondary trabeculae but are now partially replaced by several small roundish holes filled with sediment during fossilization. In Cru-3, the medullary region is displaced posteriorly and somewhat laterally, leaving the anterior and medial bone side with the thickest primary cortex. In Cru-6, the medullary region is centered, and the primary cortex is of more or less even thickness around the cross section.

### Ontogenetic Variation and Taxonomical Implications

On the basis of morphology and size, the sampled humeri represent one ontogenetic series (Table 1, Table S1). However, the two smallest humeri sampled (MDE C3-977, MDE C3-270; Table 1, Table S1) show clear differences in bone tissue and HOS. Humeri MDE C3-977 and MDE C3-270 show a low but certain amount of woven bone matrix in the scaffolding of the fibrolamellar complex, both are well laminar organized, and they lack any growth marks (Fig. 4A, B). They show no secondary osteons in their cortex.

The larger humeri assigned to *A. atacis* (Table 1, Table S1) show a rather uniform histological picture, because they are largely remodeled by secondary osteons (Table S1).

**Figure 7:**
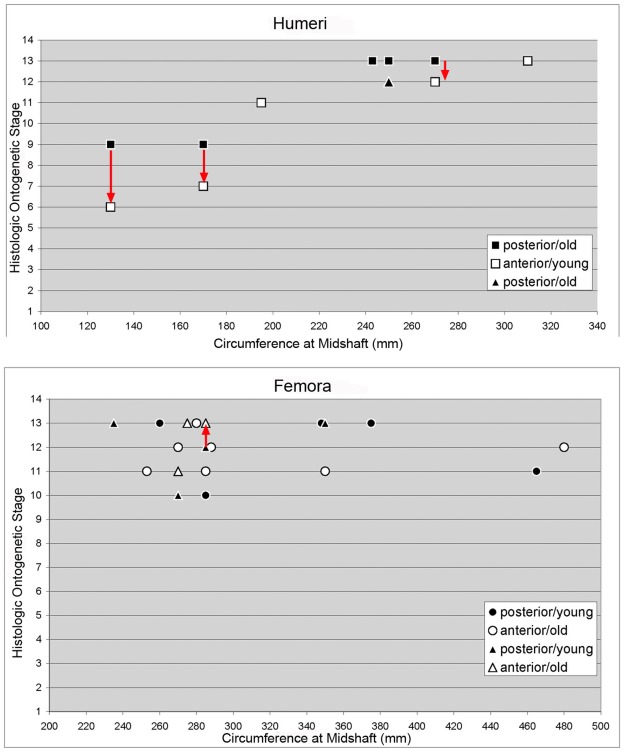
HOS of *Ampelosaurus atacis* from Bellevue locality (MDE C3) and from titanosaurs north of Narbonne (Cru) plotted against bone circumference at midshaft. Circumference at midshaft (mm) versus histological ontogenetic stage (HOS) from the posterior and the anterior bone side. Note that the posterior side in the humerus is a later stage than the anterior one (the standard sampling location) and that the anterior side of femur is older than the posterior side (the standard sampling location). Red arrows connect the anterior and posterior side of the same bone (from old to young). The rectangles mark the samples form *A. atacis* from Bellevue locality. The triangles mark the samples from titanosaurs north of Narbonne (Cru).

Based on the maximum femur length for *Ampelosaurus* of around 1100 mm, the femur sample includes half-sized individuals (min. sampled femur length is 630 mm; Table 1, Table S1). However, the histology of femora does not show a large range of HOS (Table 3, Table S1) but can be divided into two groups: some larger femora are not yet completely remodeled, but other smaller femora are (Fig. 5, 7, Table 3, Table S1). This disparity must not represent different taxa but could instead also indicate sexual dimorphism or developmental plasticity [31].

### Modified Histological Ontogenetic Stages (HOS) for *Ampelosaurus*

Differences in bone histology of *A. atacis* compared to basal neosauropods limit the application of the HOS of Klein and Sander [6], necessitating modifications to accommodate *Ampelosaurus*. Accordingly, we redefine bone tissue types C to F of Klein and Sander [6] for *Ampelosaurus* (Table 3, Fig. 7) to improve comparability with the HOS of more basal Diplodocoidea and basal Macronaria.

Type A and type B bone tissues are not known for *Ampelosaurus*. Assuming that humeri MDE C3-977 and MDE C3-270 are juveniles of *Ampelosaurus*, the definition of type C bone tissue is largely the same in *Ampelosaurus* as in other sauropods [6], except for a low amount of woven bone matrix in the scaffolding of the fibrolamellar complex. Instead parallel-fibered matrix and lamellar bone matrix builds most of the scaffolding here. Type D bone tissue of *Ampelosaurus* differs in nearly all aspects from that of Diplodocoidea and basal Macronaria. The scaffolding of the fibrolamellar complex is solely built of parallel-fibered matrix and lamellar bone matrix, resulting in much more highly organized primary bone tissue. There are no growth marks in type D bone tissue of *Ampelosaurus*, and secondary osteons are absent throughout the primary cortex (Fig. 4A, B). Type E bone tissue of *Ampelosaurus* is characterized by a moderate vascular density. The scaffolding of the fibrolamellar complex shows nearly exclusively lamellar bone matrix. However, the primary cortex still does not show any growth marks or secondary osteons. Type F bone tissue type of *Ampelosaurus* shows a generally lower vascular density (when compared to type E bone tissue type), but in some samples there is still a moderate vascular density (Fig. 4C). Primary osteons are still immature, and the framework of the fibrolamellar complex consists exclusively of lamellar bone matrix. In type F bone tissue of Diplodocoidea and basal Macronaria, a change in bone tissue type from the fibrolamellar complex to lamellar zonal bone tissue is initiated, ultimately forming an EFS (sensu Cormarck [19]). Neither an EFS nor any other growth marks occur in any bone tissues of specimens of *Ampelosaurus*. Remodeling by secondary osteons does not occur before type F bone tissue in *Ampelosaurus*. In Diplodocoidea and basal Macronaria, remodeling already starts in type D bone tissue, but the remodeling is not very dense before some type E bone tissue is deposited. Whereas in Diplodocoidea and basal Macronaria secondary osteons are initially scattered and become more and more dense during ontogeny, secondary osteons appear very suddenly in *Ampelosaurus* and from dense Haversian bone from their first appearance onwards. All *Ampelosaurus* samples show this sudden dense remodeling except for two femur samples (Cru-3; MDE C3-638). In these samples, remodeling is more moderate, with scattered or dense secondary osteons only rarely reaching the middle of cortex. Type G bone tissue in *Ampelosaurus* means a cortex completely remodeled by secondary osteons, as in basal Neosauropoda.

### Long Bone Histology of Alamosaurus Sanjuanensis

*Alamosaurus sanjuanensis* shows the laminar fibrolamellar bone tissue typical for basal neosauropods and macronarians with a scaffolding of woven bone matrix. Its cortical histology shows the ontogenetically different bone tissue types which meet the definition of the HOS as described by Klein and Sander [6] (Fig. 3A). The primary bone tissue of *Alamosaurus* has a much woven bone matrix in early ontogenetic stages. In late ontogenetic stages the amount of parallel-fibered and lamellar matrix increases successively in the scaffolding of the fibrolamellar bone of *Alamosaurus*. Humerus TMM uncataloged (1350 mm) has in its outermost cortex two LAGs deposited accompanied by a change from fibrolamellar bone to avascular lamellar bone tissue, which may represent an incipient EFS (Fig. 3B).

## Discussion

### Implications of Histology for Life History and Growth Pattern

The peculiar histology of the *Ampelosaurus* primary cortical bone and the modifications of the HOS as described here raises the question what we can deduce about patterns of growth and life history in this taxon. *Ampelosaurus* seems to have grown in a way similar to more basal sauropods (although more slowly, as evidenced by the already very highly organized bone tissue before sexual maturity is reached). After this point in ontogeny, bone microstructure of *Ampelosaurus* differs greatly from that of more basal Sauropoda. Bone tissue types D to E, representing this phase in ontogeny, are characterized by a much higher organization of primary bone and therefore presumably a lower apposition rate. However, this highly organized primary bone is inconsistent with the absence of growth marks and the relatively extensive vascularization, indicating that growth continued slowly but uninterrupted. Although Diplodocoidea and basal Macronaria also show an increase in bone tissue organization ( = decrease in growth rate) around the presumed onset of sexual maturity, it is much more gradual and this phase seems to have lasted longer than in *Ampelosaurus*. The process of remodeling by secondary osteons, which in other sauropods started around sexual maturity [6], is clearly delayed in *Ampelosaurus*. The period of growth after sexual maturity seems to be shortened in *Ampelosaurus* and is dominated by remodeling instead of fast appositional growth.

A very interesting and not yet understood fact is that most of the *Ampelosaurus* samples are dominated by type G bone tissue but do not represent the largest known individuals (Le Loeuff pers. obs.). Thus, these bones presumably do not represent fully grown (i.e., skeletally mature) individuals, which is also consistent with the lack of an EFS. This is quite different from the condition in Diplodocoidea and basal Macronaria, in which type G bone tissue only occurs after an EFS was deposited and only in the largest specimens.

### *Ampelosaurus* Histology Compared with other Titanosaurs

The observation that the cortical bone histology of *Ampelosaurus* is distinct from that of more basal, non-titanosaurian sauropods raises the question of how *Ampelosaurus* histology compares to that of other titanosaurs (as reviewed in the introductory section of this study) and how features of long bone histology relate to body size.

The largest titanosaur for which comprehensive histological information is available is *Alamosaurus sanjuanensis* (Fig. 1). In bone dimension and mass this animal rivaled large diplodocoids and large basal macronarians [32]. *Alamosaurus* also shows a similar long bone histology to the non-titanosaurian sauropods ([7], this study), and the HOS erected for basal neosauropods are perfectly applicable to *Alamosaurus* long bones (Fig. 3, Table 2).

In early ontogenetic stages, the long bones of the basal titanosaur *Phuwiangosaurus sirindhornae* (Fig. 1) show a normal amount of woven bone matrix in the fibrolamellar complex. However, the scaffolding of long bone tissue of adult (sexually mature) *Phuwiangosaurus* is dominated by parallel-fibered bone matrix and is characterized by the absence of woven bone matrix, similar to *Ampelosaurus*. Contrary to *Ampelosaurus*, *Phuwiangosaurus* has developed growth marks in the outer cortex. The lack of an EFS in *Phuwiangosaurus* is most probably a sampling artifact, because no fully grown individuals were sampled, and probably does not represent a true histological character of this taxon. An additional argument that the EFS simply was not sampled is the fact of less remodeling in *Phuwiangosaurus* than in other taxa. In its humeri, *Phuwiangosaurus* shows a similar remodeling pattern as basal neosauropods starting with scattered secondary osteons. *Phuwiangosaurus* femora show only little remodeling at all, even in late HOS. Phylogenetic body size optimizations suggest *Phuwiangosaurus* has somewhat reduced its body size (Fig. 1) compared to the stem line ancestor, and long bone histology suggests that this body size reduction evolved via a reduction in growth rate.

In the case of *Lirainosaurus astibiae*, phylogenetic optimization of body size (Fig. 1) indicates that this taxon is part of a clade of small-bodied titanosaurs. *Lirainosaurus* and *Ampelosaurus* share a high amount of parallel-fibered and lamellar bone matrix, but unlike *Ampelosaurus*, *Lirainosaurus* still has woven bone matrix included in the fibrolamellar complex [12]. The two taxa also share strong remodeling by secondary osteons. Company [12] stated that remodeling was initiated early in the ontogeny of *Lirainosaurus*, whereas we observed that remodeling in *Ampelosaurus* does not start before HOS 9/10 (Table S1), a stage which is interpreted as already representing adult (sexual mature) individuals. Although *Lirainosaurus* and *Ampelosaurus* share the absence of an EFS, they differ in the presence of growth marks in the primary cortex of *Lirainosaurus*. *Lirainosaurus* also has a clearly defined medullary cavity which is not the case in *Ampelosaurus*.

The island dwarf *Magyarosaurus dacus* experienced a dramatic evolutionary body size reduction (Fig. 1). However, among the taxa compared here, *Magyarosaurus* and *Ampelosaurus* are most similar. Both share dense and extensive cortical remodeling by secondary osteons which leads to a complete remodeling of the primary cortex long before skeletal maturity is reached. This intensive remodeling right up to the outer bone surface seems to be the reason for the absence of an EFS in both taxa. *Ampelosaurus* and *Magyarosaurus* differ in the timing of the onset of that remodeling process, which seems to start in *Magyarosaurus* much earlier in ontogeny [13] than in *Ampelosaurus* (see above). However, one could argue that the extensive remodeling by secondary osteons observed in *Ampelosaurus* was carried to extremes in *Magyarosaurus.* Differences between *Magyarosaurus* and *Ampelosaurus* concern the primary bone tissue organization. In *Magyarosaurus* the framework consists mainly of parallel-fibered bone matrix, but in *Ampelosaurus* it consists of lamellar bone matrix. Thus, the degree of bone crystallite organization is higher in *Ampelosaurus* than in *Magyarosaurus* (in which it is already higher than in non-titanosaurian sauropods).

### Modified Laminar Fibrolamellar Bone

The generally accepted definition of fibrolamellar compact bone [29] calls for a scaffolding of woven bone matrix filled in by primary osteons. The fibrolamellar complex in the long bones of *Phuwiangosaurus, Ampelosaurus*, *Lirainosaurus*, and *Magyarosaurus* differs from this definition and is also contrary to the condition in non-titanosaurian sauropods and in *Alamosaurus*. While all of these titanosaurs show the typical laminar vascular organization of sauropod primary cortical bone, the scaffolding in the fibrolamellar complex is laid down as parallel-fibered or lamellar bone instead of woven bone, at least in later ontogenetic stages. It should be noted that high amounts of parallel-fibered and lamellar bone matrix in the scaffolding should not be confused with particularly well developed primary osteons that would have developed only after the deposition of the scaffolding. Neither do we think it is likely that the primary cortical bone of *Ampelosaurus* is the result of a complete reduction of the scaffolding leading to bone formation by primary osteons only. If this were the case, we should not observe any parallel-fibered matrix at all because osteonal deposition is exclusively lamellar. The question thus arises if the primary cortical tissue of *Phuwiangosaurus, Ampelosaurus*, *Lirainosaurus*, and *Magyarosaurus* still should be called fibrolamellar bone.

To describe this peculiar primary cortical bone tissue of *Phuwiangosaurus, Ampelosaurus*, *Lirainosaurus*, and *Magyarosaurus*, we here introduce a new term, “modified laminar bone” (MLB). Based on accepted sauropod phylogenies (e.g., [33], see also Fig. 1), MLB is clearly a derived bone tissue type. Its structure also indicates that it must have evolved from the laminar fibrolamellar bone tissue typical for non-titanosaurian sauropods and *Alamosaurus* (e.g., [4], [6], [7], [16], [17], [21]) by simply laying down the scaffolding as parallel- fibered or lamellar bone matrix instead of woven bone matrix.

Differences between titanosaur bone tissue and that of more basal sauropods were already discussed by Company [12], who raised the question whether titanosaurs have “special titanosaur histology”. However, he also included the large titanosaur *Alamosaurus* in the “special titanosaur histology”, a hypothesis that clearly contradicts our own and previously published observations [7]. Thus MLB is currently only known from medium-sized and small titanosaurs. As usual for describing bone histological features, the main terminological problem is that the transition from laminar fibrolamellar complex with a scaffolding of woven bone matrix to MLB with a scaffolding largely without woven bone matrix is gradual and a quantitive definition cannot be given at the moment.

Application of Amprino’s rule [8] and general principles of biological and physiological organization suggests that MLB had significantly lower apposition rate than regular laminar bone. The initially deposited framework, with a high amount of parallel-fibered or even lamellar bone matrix in MLB could not have been deposited as fast as with woven bone matrix. This also suggests that the overall growth rate of titanosaurs showing MLB was significantly lower than that of neosauropods having grown with normal fibrolamellar bone. A test for this hypothesis is offered by the extinct insular bovid *Myotragus balearicus* [34] which grew much more slowly than similar-sized mainland bovids, as evidenced by growth mark counts. *Myotragus* also shows a complete loss of woven bone matrix from its cortex. However, illustrations and descriptions of its cortical histology are insufficient for determining whether it retained the laminar organization seen in mainland bovids [35], and whether its tissue thus is MLB.

### Growth Marks in Titanosaurs

Growth marks being generally rare in sauropods or appearing only late in ontogeny, if at all [4], is contrary to the condition in early sauropodomorphs (e.g., *Plateosaurus* and *Massospondylus*) and the sister group to Sauropodomorpha, Theropoda [1], [2]. The absence of growth marks throughout most of sauropod ontogeny is problematic because it hampers the construction of growth curves and estimates of absolute bone apposition rates [4], [36]. On the other hand, the lack of growth marks is suggestive of fast and uninterrupted growth in sauropods, which supports the hypothesis that they achieved large body size through the heterochronic process of acceleration [18].

The deposition of growth marks in the outer cortex of adult *Phuwiangosaurus* and in the middle to outer cortex of adult *Lirainosaurus* long bones starts in young adult individuals and therefore conforms to the typical pattern in sauropods. However, the more regular occurrence and higher number of growth marks in *Lirainosaurus* when compared to non-titanosaurian sauropods [6] suggests that this titanosaur, in addition to its reduced growth rate (achieved by a higher organized bone tissue), slowed growth by regular cessations in growth. Conversely, one could argue that growth in *Lirainosaurus* was slow enough so that growth marks were deposited.

*Magyarosaurus* shows LAGs only in two samples, and *Ampelosaurus* does not show any growth marks in any bone sample. This is rather inconsistent with the apparently reduced growth rate and high level of bone organization seen in both taxa, features which are often associated with the deposition of growth marks. The most likely explanations for the lack of growth marks in *Magyarosaurus* and *Ampelosaurus* are: (1) the process of remodeling by secondary osteons was so fast that the primary cortex was already remodeled before any growth marks could have been deposited and (2) existing growth marks were obliterated by remodeling shortly after they were deposited. In both cases it is clear that the remodeling process in *Ampelosaurus* and *Magyarosaurus* is much faster than in *Phuwiangosaurus* and *Lirainosaurus* or any other sauropod.

### The Lack of an EFS in Titanosaurs

Based on the observations of extant mammals by Cormack [19], skeletal maturity of an individual was reached in sauropods when an EFS developed. *Lirainosaurus*, *Magyarosaurus*, and *Ampelosaurus* do not show an EFS. Company [12] had discussed several hypotheses explaining the lack of an EFS in *Lirainosaurus* such as: an indeterminate growth strategy, remodeling by secondary osteons, or the sampled individuals were still actively growing (despite strong remodeling). However, he finally concluded that none of these hypotheses fit and that the lack of an EFS is a real histological feature of *Lirainosaurus* and possibly other titanosaurs as well [12]. Stein et al. [13] also offered various explanations for the lack of an EFS in *Magyarosaurus*: the EFS was already obliterated by secondary osteons, an EFS was generally not deposited, or the outer surface of the bones was incompletely preserved.

The absence of an EFS in *Ampelosaurus* is best explained with arguments similar to those above. The fact that the largest femora and humeri of *Ampelosaurus* are around 20% larger than the largest bones of *Ampelosaurus* sampled here may explain the lack of an EFS in this taxon as an an artifact of incomplete sampling. However, even in smaller bones of *Ampelosaurus*, remodeling by secondary osteons is so fast and intense that the entire primary bone tissue is already remodeled (Table S1), although these bones do not come from fully grown individuals. Thus, an EFS in *Ampelosaurus* could simply have been obliterated by the fast remodeling process (as suggested for *Magyarosaurus* by Stein et al. [13]). This would mean that the deposition of primary bone is closely followed by the remodeling process and, therefore, primary bone tissue is no longer visible. This hypothesis is supported by the observation that in humeri, the anterior bone side always shows a “younger” bone tissue than the posterior bone side and the other way around in femora (see above and SI). Thus, several samples still have remains of primary cortex without an EFS developed at one bone side whereas the other bone side is already completely remodeled (Table S1).

### Patterns of Remodeling by Secondary Osteons

Remodeling of primary bone tissue by secondary osteons, culminating in dense Haversian bone, is widespread in large-bodied Dinosauria and Mammalia [2], [29]. However, the meaning of this cortical remodeling process is not well understood. Several, not mutually exclusive, hypotheses explain the occurrence of secondary osteons: healing of fatigue microcracks, removal of dead tissue, remobilization of bone mineral, and simply a time-dependant process (see Currey [37] and Sander et al. [4] for a good review). A commonly cited hypothesis, that Haversian remodeling strengthens the bone, seems unlikely because Haversian bone is less strong than primary bone, at least under a bending load [37].

Klein et al. [11] hypothesized that the co-occurrence of intense remodeling and high amounts of parallel-fibered bone in *Phuwiangosaurus* may indicate that remodeling in sauropods progresses outwards at a constant rate independent of the apposition rate of the primary tissue. If apposition rate is comparatively low, as in the MLB of the titanosaurs studied here, remodeling will affect relatively more of the primary bone than in faster growing non-titanosaurian sauropods and *Alamosaurus*. Similarly, in *Ampelosaurus* and *Magyarosaurus*, the apposition front depositing primary bone tissue obviously was overrun by the process of remodeling. Additionally, in *Ampelosaurus*, the remodeling process must have been extremely fast, because it started only late in ontogeny but reached the bone surface quickly.

The argument of Company [12] that “secondary remodeling took place during pauses in periosteal bone deposition, or gradually, as the periosteal growth rate slowed down” was already falsified by the remodeling pattern in Diplodocoidea and basal Macronaria, which clearly show that remodeling takes place independently of periosteal growth [4], [6], [16]. Also, Company’s hypothesis that secondary reconstruction “equaled or even exceeded the energy requirements for deposition of primary bone” [12] is unlikely because resorption and re-deposition takes place very locally and no additional bone tissue is added; only existing tissue is remodeled. Therefore, rather the contrary is likely, and secondary bone seems to require less energy than the deposition of primary bone because no new materials need to be added to the tissue.

A relationship between the degree of secondary osteon development and ontogeny was already pointed out by Sander [16] and explored more in depth by Klein and Sander [6] and Sander et al. [4]. Among other histological features, we used the occurrence and density of secondary osteons for the definition of the histological ontogenetic stages for Diplodocoidea and basal Macronaria. However, the sudden onset of remodeling seen in *Ampelosaurus* has not been observed before in sauropods. A possible explanation involves the hormonal onset of sexual maturity. More explicitly, secondary osteon formation may possibly have been triggered or controlled by hormones which initiate sexual maturity, because bone metabolism in mammals is greatly influenced by hormones [37]. However, this hypothesis fails to explain why the onset of sexual maturity is more sudden in *Ampelosaurus* than in other sauropods.

### Evolutionary Origins of Reduced Growth Rates in Titanosaurs

In order to understand the diversity of histological patterns and growth rate reduction seen in some titanosaurs, a look at the body size of the taxa and possible effects of island dwarfing is needed. Beside the similar size, *Alamosaurus* (FL  = 1610 mm [36]) and basal neosauropods share a uniform long bone histology and show no indication for reduced growth rates. Patterns divergent from typical sauropod histology are seen in the two small titanosaurs examined or reviewed for this study and in the normal-sized *Ampelosaurus* and *Phuwiangosaurus*. Because the rate of deposition of the bone matrix determines the rate of size increase of the bone, the high amount of parallel-fibered and lamellar bone matrix in the early ontogenetic stages of *Phuwiangosaurus, Ampelosaurus*, *Lirainosaurus*, and *Magyarosaurus* implies a low rate of bone deposition and therefore a lower growth rate for these titanosaurs compared to non-titanosaurian sauropods and *Alamosaurus*.

Together with other histological peculiarities, the reduced growth rate and body size of *Magyarosaurus* (FL  = 540 mm, [13]) have been interpreted as an adaptation to limited resources on an island ([13], see also Sander et al. [20] on *Europasaurus*). This raises the question whether resources were limited for the other titanosaur species (except *Alamosaurus*) as well. Both *Ampelosaurus* (FL = ca. 1100 mm, MDE C3-02-172) and *Lirainosaurus* (FL  = 686 mm, [23]) inhabited large islands of the Late Cretaceous southwestern European archipelago [38], [39], the former the southern French island in the Campanian-Maastrichtian and the latter the Iberian island in the Campanian. While *Lirainosaurus* may have responded to the resource limitations by a reduction in body size (although body size optimizaton suggests otherwise) and a decrease in growth rate, the larger *Ampelosaurus* only decreased its growth rate.

An insular habitat limiting resources can be excluded in the case of the relatively large-bodied *Phuwiangosaurus* (FL  = 1250 mm [23]) that inhabited the Indochina peninsula which remained fully connected to the East Asian land mass throughout the Cretaceous [40]. Thus, while there may well be a distinctive island signal in the decrease in body size and/or growth rate in titanosaurian bone histology (*Ampelosaurus*, *Lirainosaurus*, and *Magyarosaurus*), other adaptive pressures must also have been able to produce reduced growth rates in titanosaurs, notably *Phuwiangosaurus*. A more complete understanding of this evolutionary phenomenon and the evolution of MLB must await a comprehensive sampling of titanosaur bone histology, especially of the South American species, combined with patterns of body size evolution in titanosaurs [13].

### Conclusions

Based on a detailed histological analysis of the cortical long bone histology of a growth series of the derived titanosaur *Ampelosaurus*, we review and discuss titanosaurian bone histology. Although taxon sampling is limited (*Alamosaurus*, *Ampelosaurus*, *Lirainosaurus*, *Magyarosaurus*, *Phuwiangosaurus*), four out of the five taxa (*Alamosaurus* being the exception) show a peculiar primary cortical tissue, here termed *Modified Laminar Bone* (MLB), that evolved from the standard laminar fibrolamellar bone (FLB) of sauropods. During the evolution of MLB, the laminar organization of the vascular network was retained, but only the initial scaffolding of bone deposition in early ontogenetic stages contains a low amount of woven bone matrix (as in typical FLB). During most of ontogeny the scaffolding consists of highly organized parallel-fibered bone matrix grading into lamellar bone matrix instead of woven bone matrix. The gradual infill of the vascular canal by lamellar bone, i.e. the formation of primary osteons, in FLB was retained in MLB. Currently, MLB is unique to the four titanosaur taxa studied here, but it also may have evolved in the extinct island bovid mammal *Myotragus*. MLB and patterns of remodeling also necessitated the modification of histological ontogenetic stages (HOS) of more basal sauropods for usage in *Ampelosaurus*.

MLB unequivocally indicates a greatly reduced cortical apposition rate compared to laminar FLB, and thus a greatly reduced growth rate of the titanosaurs that show it. This reduction is difficult to quantify because of the lack of growth marks seen in most titanosaur taxa with MLB. While the drastic growth rate reduction documented by MLB may have evolved as a response to resource limitations on an island, this hypothesis is only satisfactory for the island dwarf *Magyarosaurus* [13] and the possible island dwarf *Lirainosaurus*. Paleogeography suggests that the non-dwarfed *Ampelosaurus* may also have responded with MLB to resource limitations, but this is not the case in *Phuwiangosaurus*. Clearly, a better understanding of MLB in titanosaurs must await a more comprehensive taxon sampling.

## Supporting Information

Table S1 **Histological description of sampled bones of** ***Ampelosaurus atacis*** **from Bellevue locality (MDE C3) and the titanosaurs from north of Narbonne (Cru).** The bones are listed by midshaft circumference (see Table 1). Abbreviations: FLB = fibrolamellar bone tissue; ic = inner cortex; lb = lamellar bone matrix; mc = medullary cavity; oc = outer cortex; pfb = parallel-fibered bone matrix; po = primary osteons; wb = woven bone matrix.
(DOC)
