# Supplementary material for: Modified Laminar Bone in Ampelosaurus atacis and Other Titanosaurs (Sauropoda): Implications for Life History and Physiology
Source: PLoS One. 2012 May 16;7(5):e36907. doi: 10.1371/journal.pone.0036907 (PMC3353997; doi:10.1371/journal.pone.0036907)
Supplement: Table S1 — Histological description of sampled bones of Ampelosaurus atacis from Bellevue locality (MDE C3) and the titanosaurs from north of Narbonne (Cru). The bones are listed by midshaft circumference (see Table 1). Abbreviations: FLB = fibrolamellar bone tissue; ic = inner cortex; lb = lamellar bone matrix; mc = medullary cavity; oc = outer cortex; pfb = parallel-fibered bone matrix; po = primary osteons; wb = woven bone matrix. (DOC) [file pone.0036907.s001.doc]

| **Humeri** | **Primary bone tissue** | **Vascular density/ organization** | **Growth marks**  **/EFS** | **Secondary osteons** | **size of mc** | **comment** |
| --- | --- | --- | --- | --- | --- | --- |
| **C3-977** |  |  |  |  |  |  |
| anterior | FLB with high amount of lb but also wb, well developed po | high,  laminar | none/  no EFS | none | 75% of the anterior-posterior diameter |  |
| posterior | FLB with high amount of lb, no wb, well developed po | moderate,  laminar | none/  no EFS | few scattered | 70 to 80% of the lateral-medial diameter |  |
| **C3-270** |  |  |  |  |  | vascular canals diagenetically widened |
| anterior | FLB with high amount of lb and no wb; poorly developed po | ic: high  oc: moderate,  roughly laminar | none/  no EFS | none | 78% of the anterior-posterior diameter |  |
| posterior | FLB with high amount of lb and no wb, poorly developed po | ic: moderate  oc: low,  laminar | none/  no EFS | none | 73% of the lateral-medial diameter |  |
| **C3-1506**  anterior | no wb, high amount of pfb, well developed nearly closed po | moderate,  laminar | none/  no EFS | ic: dense  oc: scattered |  |  |
| **C3-175**  posterior | -- | -- | none/  no EFS | nearly completely remodeled |  |  |
| **C3-602**  posterior | -- | -- | none/  no EFS | completely remodeled |  |  |
| **Cru-1723**  anterior | only lb | moderate,  laminar | none/  no EFS | relatively dense through entire cortex |  |  |
| **C3-238** |  |  |  |  |  | bone surface is partially incomplete |
| anterior | FLB with high amount of lb, no wb, well developed po | moderate,  laminar | none/  no EFS | ic: dense  oc: scattered | 61% of the anterior-posterior diameter |  |
| posterior | -- | -- | none/  no EFS | completely remodeled | 43 % of the lateral-medial diameter |  |
| **C3-1189**  anterior | lb | -- | none/  no EFS | completely remodeled |  |  |
| **Cru-1**  posterior | -- | -- | none/  no EFS | completely remodeled |  |  |
| **Femora** |  |  |  |  |  |  |
| **Cru-2**  posterior | -- | -- | none/  no EFS | completely remodeled |  |  |
| **C3-1182**  anterior | FLB consisting mainly of pfb, only less lb, no wb , poorly developed po | moderate,  laminar | none/  no EFS | ic: dense  oc: scattered |  |  |
| **C3-708**  posterior | -- | -- | none/  no EFS | completely remodeled |  |  |
| **C3-203**  anterior | FLB consists mainly of lb, poorly developed po | -- | none/  no EFS | nearly completely remodeled except for outermost cortex |  |  |
| **Cru-3** |  |  |  |  |  |  |
| anterior | lb, poorly developed po | low,  roughly laminar | none/  no EFS | only very few scattered | 61% of the anterior-posterior diameter |  |
| posterior | FLB with a high amount of lb,  wb only in the innermost cortex, poorly developed po | moderate,  laminar | none/  no EFS | only very few scattered | 60% of the lateral-medial diameter |  |
| **C3-527**  anterior | -- | -- | none/  no EFS | completely remodeled |  |  |
| **C3-261**  anterior | FLB consists mainly of pfb, no wb, poorly developed po | moderate to low,  laminar | none/  no EFS | ic: dense  oc: densely scattered |  |  |
| **C3-638** |  |  |  |  |  |  |
| anterior | FLB with a high amount of lb; no wb, well developed po | moderate,  laminar | none/  no EFS | ic: dense  oc: densely scattered | not measurable |  |
| posterior | FLB with a high amount of lb, no wb, well developed po | high,  laminar | none/  no EFS | only very few scattered in ic | not measurable |  |
| **Cru-4** |  |  |  |  |  |  |
| anterior | FLB with a similar amount of pfb & lb, no wb | -- | none/  no EFS | nearly completely remodeled except for outermost cortex | not measurable |  |
| posterior | FLB with a similar amount of pfb & lb, no wb, well developed po | moderate,  roughly laminar | none/  no EFS | nearly completely remodeled except for outermost cortex | not measurable |  |
| **C3-143**  median | -- | -- | none/  no EFS | completely remodeled |  |  |
| **Cru-5**  posterior | -- | -- | none/  no EFS | completely remodeled |  |  |
| **C3-1239**  anterior | FLB with high amount of lb; no wb, well developed po | moderate,  roughly laminar | none/  no EFS | ic: dense  oc: scattered |  | incomplete bone surface |
| **C3-582**  posterior | lb | -- | none/  no EFS | nearly completely remodeled | crushed osteons |  |
| **C3-78**  posterior | FLB with high amount of lb, no wb, poorly developed po  component | moderate,  roughly laminar | none/  no EFS | scattered |  |  |
| **C3-174**  anterior | FLB with high amount of pfb, less lb, no wb,  poorly developed po | low,  roughly laminar | none/  no EFS | ic: dense  oc: scattered |  |  |
| **Cru-6** |  |  |  |  |  | partially incomplete bone surface |
| anterior | -- | -- | none/  no EFS | completely remodeled | 50% of the anterior-posterior diameter |  |
| posterior | -- | -- | none/  no EFS | completely remodeled | 59% of the lateral-medial diameter |  |
